# A Diverse Fiber Mixture, Reflective of a Nutritionally Balanced Diet, Is Well Tolerated by Healthy Adults

**DOI:** 10.1016/j.cdnut.2026.107695

**Published:** 2026-04-18

**Authors:** Lotte Dopheide, Marta Kozior, Amra Buco, Jeroen van Bergenhenegouwen, Sebastian Tims, Johan de Vogel-van den Bosch, Justin Roberts, Daisy Jonkers, Louise Harvey, Ardy van Helvoort

**Affiliations:** 1 Danone Research & Innovation, Utrecht, The Netherlands; 2 Department of Gastroenterology-Hepatology, Nutrition and Translational Research in Metabolism (NUTRIM), Maastricht University, Maastricht, The Netherlands; 3 Cambridge Centre for Sport and Exercise Sciences, Faculty of Science and Engineering, Anglia Ruskin University, Cambridge, United Kingdom; 4 Department of Respiratory Medicine, NUTRIM, Maastricht University, Maastricht, The Netherlands

**Keywords:** arabinoxylan, β-glucan, pectin, resistant starch, healthy adults, gastrointestinal tolerance, in vitro fermentation, fiber gap, fiber diversity

## Abstract

**Background:**

Adequate fiber intake is associated with health benefits and reduced disease risk. It is hypothesized that consuming a mixture of fiber types with distinct (physico)chemical characteristics, reflective of those naturally present in a nutritionally balanced diet, may help bridge the known “fiber gap” in many populations. However, good gastrointestinal (GI) tolerance of such a mixture of fiber types is essential to reach adequate fiber intake.

**Objectives:**

To evaluate GI tolerance of a diverse fiber mixture in healthy adults.

**Methods:**

In an open-label, single-arm, single-center study, healthy adults (45‒69 y) consumed 6 g/d of a diverse fiber mixture (FM) (2 × 3 g/d) for 4 wk alongside their habitual diet. FM comprised arabinoxylan, β-glucan, pectin, and resistant starch in proportions similar to those typically consumed in a nutritionally balanced diet. GI tolerance symptoms were assessed weekly using the Gastrointestinal Symptom Rating Scale (GSRS), along with fecal transit type and frequency, digestion-associated quality of life [Digestion Associated Quality of Life Questionnaire (DQLQ)], compliance, safety, and dietary intake.

**Results:**

Of 41 enrolled participants, 38 participants completed the study without major protocol deviations. Changes in GSRS dimensions remained below their respective minimal important differences, a measure of clinical relevance, except for the indigestion dimension from baseline to week 1, with a least-square mean and standard error of 0.4 ± 0.2, where the upper 95% confidence interval (0.0, 0.7) reached the minimal important differences of 0.7. GSRS scores remained otherwise stable over time. Weekly fecal frequency significantly increased from baseline to week 1 with least-square mean ± standard error of 1.4 ± 0.4 and 95% confidence interval (0.5, 2.3). No significant changes were observed in fecal frequency (week 4), fecal transit type, and the digestion-associated quality of life questionnaire.

**Conclusions:**

Daily consumption of 6 g FM for 4 wk was well tolerated by healthy adults.

This trial was registered at clinicaltrials.gov as NCT06115057.

## Introduction

Strong evidence supports the importance of adequate fiber intake for gastrointestinal (GI) health [[1], [2], [3]], reduced risk for disease development like type 2 diabetes, cancer [[3], [4], [5], [6]], and metabolic health [4]; including reduced risk of obesity development, improved satiety, appetite control, and body weight management [1,3,4]. Despite this evidence, the majority of the world’s population does not reach the recommended fiber intake via their daily diet, resulting in a so-called “fiber gap” [7,8]. This “fiber gap” underlies a public health concern and warrants the need for strategies like public health education on fiber-rich diets and/or fiber fortification [[9], [10], [11]]. Both the European Food Safety Authority and WHO recommend a minimum of 25 g fiber intake per day for adults [12,13], with some local authorities recommending higher intake [7]. Barriers to adequate fiber consumption could be sensory factors, such as taste, texture, and color, of naturally fiber-rich foods, particularly whole-grain foods [14]. In addition, a lack of understanding about fiber-rich foods and their benefits [15], or GI tolerance symptoms that may occur with a sudden increase or change in fiber intake, could reduce consumer acceptance [16].

A nutritionally balanced diet includes a wide range of foods that contribute to overall fiber intake, although total fiber intake often remains insufficient [7,8]. Major dietary sources of fiber include grains, fruits, vegetables, nuts, and legumes [7,8,17], providing distinct fiber types. For example, whole-grain cereals provide arabinoxylan and β-glucan, whereas various fruits and vegetables are rich in pectin. Resistant starch and cellulose are commonly present in whole grains and legumes, whereas onions and chicory root are rich sources of inulin [7,[17], [18], [19], [20]]. Thus, in a nutritionally balanced diet, individuals typically consume a variety of fiber types, generally rich in arabinoxylan, β-glucan, pectin, and resistant starch [7,[17], [18], [19], [20]].

Fiber types differ substantially in their physicochemical (including solubility, fermentability, viscosity, and water binding capacity) and chemical characteristics (including structural complexity and diversity, linkage types, branching, monosaccharide composition, and degree of polymerization). These characteristics collectively influence their physiological functionality [1,16,[21], [22], [23]]. The health benefits of fibers are largely attributed to their fermentability in the gut [[21], [22], [23]], resulting in the production of short-chain fatty acids (SCFA) with pronounced health benefits [22]. However, rapid and/or excessive fermentation results in the production of gas [24,25], which may induce GI tolerance symptoms such as flatulence, bloating, and abdominal pain [26,27].

A recent review summarized GI tolerance levels for several fiber types when added to the habitual diet [16]. For example, daily intake of 12 g of resistant starch or 25 g of soy fiber was generally well tolerated by healthy adults, whereas only 5 g of inulin per day could lead to GI tolerance symptoms like bloating and flatulence [16]. GI tolerance symptoms can range from mild manifestations, including bloating and flatulence, to more severe symptoms like diarrhea. These symptoms often decrease over time as the gut microbiota and gut physiology adapt, with an average adaptation period of ∼2 wk [16]. Therefore, a gradual increase in fiber intake is commonly recommended. To complement human GI tolerance data, preclinical gut fermentation models, such as semi-dynamic batch fermentation models, are representative models offering a controlled approach to investigate fermentability, including SCFA and gas production from individual fibers or fiber mixtures. These models provide an early indication of potential GI tolerance issues prior to conducting human clinical studies.

Considering that most of the Western population has a “fiber gap” [7,8], fortifying diets with a mixture of diverse fibers with distinct characteristics, like those present in a nutritionally balanced diet, may be a solution to meet the fiber recommendations. In an optimal situation, this fiber mixture would be well-tolerated and contribute to the generation of SCFAs without causing excess gas formation. Thus, the aim of this study was to examine the GI tolerance of a diverse fiber mixture (FM), in proportions similar to those typically consumed in a nutritionally balanced diet, by using both in vitro and clinical approaches.

## Methods

### Fiber type selection and FM composition

Four major classes of fiber types, comprising arabinoxylan, β-glucan, pectin, and resistant starch, were combined to create the diverse fiber mixture (FM). The selected fibers were a variety of plant-based, gluten-free, naturally occurring, and (physico)chemically diverse fibers, and were incorporated in proportions similar to fiber intake in a nutritionally balanced diet [7,[17], [18], [19], [20]]. The final proportions of the FM were 5:2:2:1 for arabinoxylan, β-glucan, pectin, and resistant starch, respectively.

### In vitro fermentation

A semi-dynamic batch fermentation model was used as a representative preclinical model of the dynamic state of the human gut following fiber consumption. This model enables the quantification of fermentation metabolites, like SCFAs, and gas production. The fermentation experiment was performed in duplicate in an anaerobic chamber.

Fecal material was collected from 5 healthy adults aged 23‒48 y (2 male, 3 female), without chronic GI diseases or functional GI disorders, and who had not used antibiotics in the 4 mo preceding the donation. Fecal samples were pooled, homogenized, divided into smaller aliquots, mixed with 10% glycerol in an anaerobic chamber, and stored at ‒80°C until use.

Prior to fermentation, a pooled fecal sample was thawed and mixed with slightly modified McBain and McFarlane fermentation medium [28] at a weight ratio of 1:5 in a Falcon tube to create the fecal suspension. The fermentation medium contained buffered peptone water 3.0 g/L, yeast extract 2.5 g/L, tryptone 3.0 g/L, L-cysteine-HCl 0.4 g/L, bile salts 0.05 g/L, K_2_HPO_4_ · 3H_2_O 2.6 g/L, NaHCO_3_ 0.2 g/L, NaCl 4.5 g/L, MgSO_4_ · 7H_2_O 0.5 g/L, CaCl_2_ · 2H_2_O 0.3 g/L, FeSO_4_ · 7H_2_O 0.005 g/L. Compounds were added to demineralized water, and the pH was adjusted to 6.3 ± 0.1 with K_2_HPO_4_ or NaHCO_3_, to represent the adult intestinal environment. Fermentation medium was sterilized (121°C, 15 min) and placed in the anaerobic chamber for ≥16 h before use.

Six milliliters of fecal suspension was added to a sterile Falcon tube and mixed thoroughly with 200 mg fiber (standardized fiber content across conditions) or without fiber as a blank condition. FM was premixed into a homogeneous mixture before addition. The fecal suspensions were transferred into dialysis tubes (MWCO 500, Spectra Por) and anaerobic gas was manually removed by holding the dialysis tube from the outside, pushing the anaerobic gas out, and closing the tube. The dialysis tube was placed in a 100 mL Schott bottle filled with 100 mL buffered dialysis medium. The dialysis medium had the same composition (minus peptone water, yeast extract, tryptone, L-cysteine-HCl, and bile salts) and pH as the fermentation medium, except that it was not sterilized to prevent sediment formation. The medium was placed in the anaerobic chamber for ≥16 h before use.

The Schott bottles were closed with a lid with a flexible insert (for gas quantification) and incubated at 37°C. Dialysis medium samples were taken at t = 0 h, t = 12 h, t = 24 h, t = 48 h, and t = 72 h, and after each sample point, the dialysis medium was refreshed. Samples were stored at ‒80°C until further use. Gas production was measured directly from the Schott bottles at the same timepoints except t = 0 h. Findings for SCFA and gas production are reported for t = 72 h, as a representation of full fiber fermentation across the colon.

#### SCFA quantification

Dialysis medium samples collected at t = 72 h were centrifuged at 15,000 × *g* at 4°C for 5 min. The SCFAs acetate, propionate, and butyrate were analyzed as described before [29]. In short, a Shimadzu-GC2025 gas chromatograph with a flame ionization detector (split mode) (Shimadzu Corporation) was used, via the described protocol, a calibration curve was created to determine the SCFA concentrations. Total SCFA production was calculated as the sum of acetate, propionate, and butyrate.

#### Gas quantification

The Schott bottles were shaken thoroughly before quantifying gas volume. Gas volume was measured directly from the Schott bottles with a unit to measure pressure (West 6700 Pressure Limit Controller, West Control Solutions). A 5 mL or 30 mL syringe was connected to the unit and inserted via the flexible insert in the lid into the Schott bottles. Gas was removed until no pressure was left, and the collected volume was recorded in milliliters. The SCFA-to-gas ratio was calculated by dividing total SCFA production by total gas volume at t = 72 h.

### GI tolerance study

#### Study design

**FIGURE 1:**
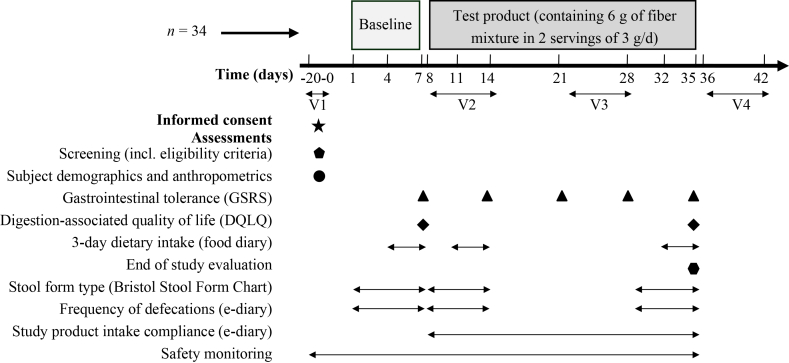
Schematic diagram of study design in healthy adults examining the effects of fiber mixture (FM) intake on gastrointestinal tolerance. DQLQ, Digestion Associated Quality of Life Questionnaire; GSRS, Gastrointestinal Symptom Rating Scale; V, visit.

This open-label, single-arm clinical study examined GI tolerance of a daily intake of 6 g FM (2 × 3 g/d) over 4 wk in healthy adults aged 45‒69 y, in addition to their habitual diet (Figure 1). The study was approved by the Stichting Beoordeling Ethiek Biomedisch Onderzoek (METC Assen, The Netherlands) on 7 September, 2023, and registered at clinicaltrials.gov (NCT06115057). The study was conducted between September 2023 and December 2023 at the Nutrition Clinical Research Unit, Danone Research & Innovation (Utrecht, The Netherlands), in accordance with International Council for Harmonisation - Good Clinical Practice (R2), the Declaration of Helsinki, and applicable Dutch laws and regulations.

#### Participants

Participants were recruited through Link2Trials (www.link2trials.com). Eligible individuals were healthy adults aged 45‒69 y with a BMI (in kg/m^2^) of 18.5‒29.9, a habitual fecal frequency of 3 stools per week to 3/d (excluding 3 loose or liquid stools per day) within 1 mo before screening, and had to be willing to comply with the study protocol requirements. The term “healthy” was defined as being in a healthy condition, considering a person’s age, in the opinion of the investigator (e.g., a person with chronic hypertension whose blood pressure was on stable medication was eligible). Key exclusion criteria included presence or history of chronic GI disease or bowel resection; medical conditions commonly/likely affecting GI function; uncontrolled chronic disease; recent surgical procedures related to the GI tract; and specified drugs and supplements. Full exclusion criteria are listed in Supplementary Table 1.

Sample size calculation was performed based on Alyousif et al. [30]. A conservative scenario was chosen for the sample size; therefore mean difference was increased from 0.01 to 0.10, standard deviation (SDs) were increased by 20% (from 0.41 to 0.49 for baseline and 0.33 to 0.40 for follow-up), and a correlation of 0.30 between the baseline and postbaseline measurements was used. Considering an α level of 0.025 with 1-sided testing and 90% power based on a paired t-test, [applying PROC POWER, SAS (SAS version 9.4_TS1M3 or higher in SAS Life Science Analytics Framework version 4.7.3 or higher) for LIN X64, SAS Institute Inc.], a sample size of 34 completers was deemed sufficient and conservative to show noninferiority with the diarrhea minimal important difference (MID) of 0.4. Assuming a dropout rate of 20%, we aimed to recruit 43 participants.

#### Study procedure

Written informed consent was obtained from all participants prior to the screening. At screening, eligibility criteria were confirmed, demographic information was collected, and participants received the study product for the full intervention period with instructions. Paper diaries were provided to record dietary intake (baseline, week 1, week 4), symptoms, medications, and supplement use (recorded when applicable); records were reviewed during onsite visits (screening, week 1, week 2, and week 4). Participants used an electronic patient-reported outcome system (Viedoc Me, version 4.77; Viedoc Technologies) to record GI tolerance (baseline and weeks 1‒4); fecal transit type and fecal frequency (baseline, week 1, week 4); digestion-associated quality of life (baseline and week 4); and FM intake compliance (weeks 1‒4) (Figure 1). Participants were encouraged to contact the site with any questions or concerns during the study.

#### Study product and compliance

FM (arabinoxylan, β-glucan, pectin, and resistant starch) was developed by Danone Research & Innovation and manufactured by Vitablend. FM was provided to the study participants as a powder supplement in a single 500 g bag, instructed to be stored in a cool and dry place. Participants were instructed to weigh the study product to 1 decimal point using provided scales and consume FM twice daily (2 × 3 g fiber per day) with solid food or dissolved in fluids (simultaneous solid food intake was required when mixed with fluids). Participants recorded their compliance with the FM intake instructions daily. Participants who had intake of <75% of the recommended dosage of the study product during the intervention were considered nonevaluable.

#### Outcomes and assessments

The primary objective was to assess if the Gastrointestinal Symptom Rating Scale (GSRS) diarrhea dimension score remained within the acceptable limit, referred to as MID, from baseline to week 4. The secondary objective was to assess if all GSRS dimensions from baseline to weeks 1‒4 were within acceptable limits. The impact of FM consumption on fecal transit type [Bristol Stool Form Scale (BSFS)], fecal frequency, and digestion-associated quality of life [Digestion Associated Quality of Life Questionnaire (DQLQ)] was assessed as an exploratory objective.

The GSRS comprises 5 dimensions (diarrhea, abdominal pain, constipation, indigestion, and reflux), rated on the 7-point Likert scale, where 1 denotes no symptoms (e.g., no discomfort), 2 minor symptoms, and 7 the most pronounced symptoms (e.g., very severe discomfort), to evaluate GI tolerance over the preceding 7 d [31,32]. MID values were used as a measure of clinically perceived relevance and were as follows: diarrhea 0.4; abdominal pain 0.6; constipation 0.6; indigestion 0.7; and reflux 0.8 [33]. Fecal transit type was assessed for each defecation using the BSFS, with scores 1‒2 classified as slow transit, 3‒5 as normal transit, and 6‒7 as fast transit [34]. Fecal frequency was derived from the BSFS and the daily answer to the question: “Did you have any stools today?” The DQLQ contains 9 statements covering dietary intake, physical and emotional well-being, social activities, and physical appearance, using a 7-point Likert scale from 0% (never) to 100% (always), to evaluate the impact of GI tolerance symptoms on quality of life over the preceding 7 d [35]. Food and fluid intake were assessed by a 3-d dietary record, recording intake using weighing scales and household measures on 3 consecutive days (1 weekend day and 2 weekdays). Dietary intake records were entered into the nutrition analysis software (Evry B.V.; desktop-based version accessed 2024) based on the Dutch Food Composition Database to calculate energy, macronutrient, fiber, and water intake.

#### Safety

Adverse events (AEs) and serious AEs and their incidence, seriousness, severity, and relatedness were recorded via participant report and site observation. Safety assessments were analyzed separately from GSRS outcomes.

### Statistical analysis

#### Analysis data sets

The full analysis set (FAS) consisted of all subjects treated, i.e., all participants who had consumed the study product. The per-protocol (PP) data set consisted of all participants from the FAS data set who completed the study without infringing compliance criteria or any major protocol deviations. The PP differed by GSRS dimension, since medication use that might result in an optimistic estimate of GSRS dimension scores was considered for exclusion from PP (Supplementary Figure 1). The PP data set is presented in the main text to obtain more conservative estimates of GI tolerance symptoms of FM consumption. The FAS outcomes are reported mostly in the supplementary material.

#### Descriptive statistics and statistical models

Descriptive statistics were summarized for all outcomes. Data are presented as mean ± SD for continuous parameters, unless noted otherwise, depending on the normality of the distribution. Data are presented as numbers and percentages for categorical variables and for missing values. For all statistical models, standard QQ-plots and histogram and scaled residual plot checks were used to assess the validity of modeling assumption.

GSRS scores, fecal transit type, fecal frequency, and DQLQ were analyzed using a mixed model for repeated measures with an unstructured variance-covariance matrix, including baseline in the outcome vector and timepoint as a repeated measure and fixed factor in the model, using 95% confidence interval (CI) to test for noninferiority. Changes in outcomes were estimated from baseline at each week and compared with the relevant MIDs (GSRS) or tested for nonzero change in the absence of MIDs (fecal transit type and frequency, DQLQ). Dietary intake changes from baseline were tested with paired t-tests, and the Wilcoxon signed rank test if ≥2 d out of 3 d were recorded per assessment week. Covariate analyses were conducted for primary and secondary outcome parameters, following the sensitivity analysis as outlined by Morris et al. [36]. The significance level was α = 0.025 for GSRS and α = 0.05 for other outcomes. For the in vitro fermentation, no statistical analyses were performed because the experiment was performed in duplicate. Fold changes were calculated for the individual fibers compared to FM.

## Results

### In vitro fermentation

**FIGURE 2:**
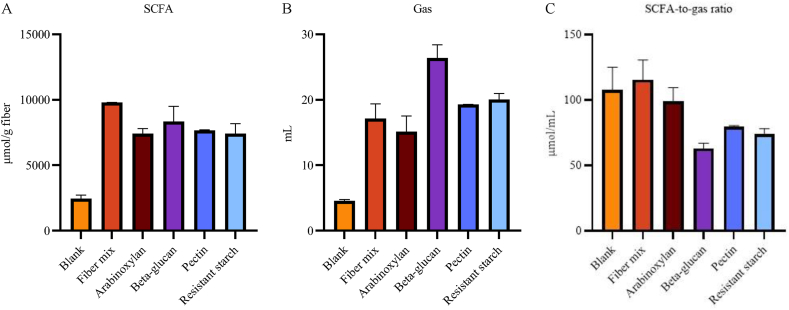
In vitro fermentation in a semi-dynamic batch fermentation model with a fecal pool of healthy adult donors for 72 h, testing blank reference control without fiber, individual fibers, and the fiber mixture thereof, standardized for fiber content of 200 mg. (A) Short-chain fatty acid (SCFA) reported as total sum of acetate, propionate, and butyrate. (B) Total gas production. (C) Calculated ratio between the production of SCFA and gas, represented as SCFA-to-gas ratio. All fibers were added at the same dose to enable like-for-like comparisons. Data are represented as mean ± SD of duplicate experiments. SD, standard deviation.

In vitro fermentation results of FM and its individual fibers are shown in Figure 2. All fibers, at a total dose of 200 mg each, increased SCFA concentrations relative to the blank control. The total SCFA production of each individual fiber was lower than that of FM, with fold changes of 0.76 for both arabinoxylan and resistant starch, 0.78 for pectin, and 0.85 for β-glucan compared with FM (Figure 2A). Acetate was the most abundant SCFA for all fibers, followed by propionate or butyrate depending on the fiber type (Supplementary Table 2).

Gas production increased for all fibers relative to the blank control (Figure 2B). The total gas production of most individual fibers was higher than that of FM, with fold changes of 1.54 for β-glucan, 1.13 for pectin, and 1.17 for resistant starch, whereas arabinoxylan produced slightly less gas (0.88-fold change) compared with FM (Figure 2B).

The SCFA-to-gas ratio was higher for FM than all individual fibers, with respective fold change of 0.86 for arabinoxylan, 0.55 for β-glucan, 0.69 for pectin, and 0.64 for resistant starch compared with FM (Figure 2C). Although the blank showed a comparable SCFA-to-gas ratio as FM (0.94-fold change), its total SCFA production remained lower (Figure 2A).

### GI tolerance study

#### Participant characteristics

The participant flowchart is presented in Supplementary Figure 1. Of the 43 enrolled participants, 2 withdrew prior to baseline, 2 withdrew during week 1 because of unrelated illness, and probably FM-related moderate constipation. For the PP data set, 1 further participant was excluded because of study product intake prior to the start of the study, resulting in 38 participants. Additionally, 2 participants were excluded from specific GSRS dimensions, prior to analysis, due to concomitant medication use that might reduce possible GI symptoms (1 participant was excluded for the reflux dimension due to omeprazole use, and 2 from the constipation dimension due to bisacodyl or magnesium citrate use). Baseline participant demographics for the FAS data set are shown in Table 1. The demographics of the PP data set did not differ from FAS.

**TABLE 1:** Participant demographics and baseline characteristics of the full analysis set^1^

Characteristics	Participants (*n* = 41)
Sex (*n*)	
Male	11
Female	30
Age, y	54.2 ± 6.3
Body weight, kg	73.1 ± 13.0
BMI, kg/m^2^	24.7 ± 2.8

1 Values are mean ± SD and absolute number for sex.

#### Product compliance and dietary intake

Median (IQR) self-reported product intake compliance over the full study duration was 100.0% (99.6‒100.0) in the PP data set and 100.0% (98.7‒100.0) in the FAS data set. Dietary intake showed no significant changes in energy, macronutrients, fiber (without FM), or water intake between baseline and week 1 or week 4 (Table 2). Mean fiber intake without FM was close to the recommended intake of 25 g/d at all timepoints. When categorizing the participants by habitual fiber consumption below or meeting recommended fiber intake, 63.2%, 56.8%, and 57.9% of the participants had fiber intake below recommended levels of 25 g/d for baseline, week 1, and week 4, respectively (Table 2). Inclusion of the 6 g/d FM supplementation resulted in 10 additional participants meeting the recommendations at week 1 and 7 additional participants at week 4.

**TABLE 2:** Dietary intake assessment of the participants without fiber mixture intake, recorded with 3-d food diaries, of the per-protocol data set^1^

Dietary intake	Baseline	Week 1	Week 4
*n* (missing)^2^	38 (0)	37 (1)	38 (0)
Energy, kcal/d	1948 ± 529	1980 ± 533	2006 ± 514
Protein, g/d	76.8 ± 23.0	78.9 ± 24.7	79.9 ± 23.0
Carbohydrate, g/d	195.5 ± 66.3	202.6 ± 64.5	200.6 ± 57.9
Fat, g/d	84.1 ± 29.6	82.7 ± 26.8	87.3 ± 31.8
Fiber^3^, g/d	24.0 ± 10.3	24.7 ± 8.5	24.4 ± 8.8
Min, max, g/d	10.1, 52.5	10.2, 49.7	11.8, 49.3
Median (IQR)	21.3 (17.3‒26.8)	23.0 (18.5‒29.5)	23.3 (16.9‒29.7)
Below recommendation^4^, *n*	24	21	22
Fiber, g/d	18.0 ± 3.3	18.8 ± 4.0	18.2 ± 3.8
Meeting recommendation^4^, *n*	14	16	16
Fiber, g/d	34.3 ± 10.0	32.3 ± 6.4	32.8 ± 6.4
Water intake, L/d	2.5 ± 0.8	2.5 ± 0.8	2.3 ± 0.8

Abbreviations: FM, fiber mixture; IQR, interquartile range; min, minimum; max, maximum.

1 Values are mean ± SD or absolute number of participants.

2 Participants were included in the analysis if they completed minimum of 2 full days of dietary intake.

3 Fiber intake from the habitual diet, excluding FM consumption.

4 Recommended fiber intake of 25 g/d, based on European Food Safety Authority and WHO guidelines.

#### Gastrointestinal Symptom Rating Scale

**FIGURE 3:**
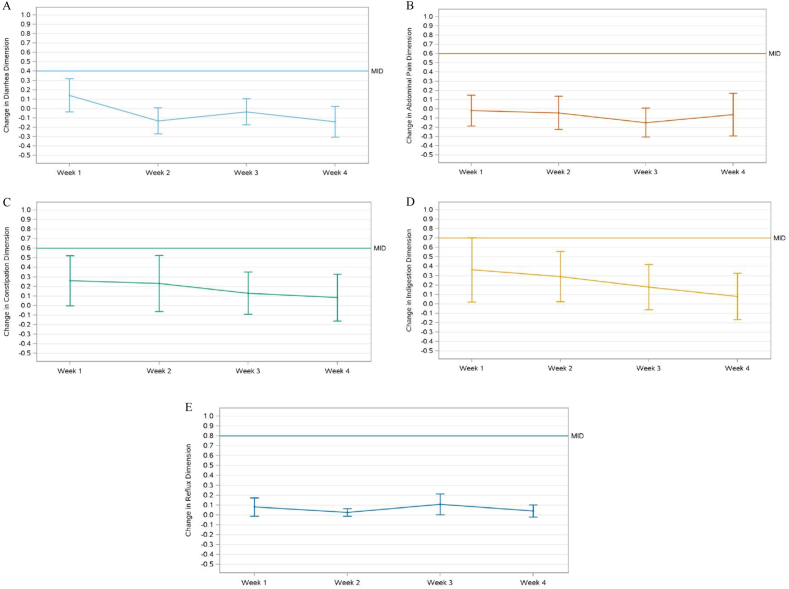
Change in Gastrointestinal Symptom Rating Scale scores from baseline, presented as predicted changes with 95% confidence interval, derived from a mixed model for repeated measures, of the per-protocol data set for the following dimensions: (A) diarrhea, (B) abdominal pain, (C) constipation, (D) indigestion, (E) reflux. The horizontal line represents the minimal important difference (MID), a measure of clinical relevance. Sample size per dimension: diarrhea *n* = 38, abdominal pain *n* = 38, constipation *n* = 36, indigestion *n* = 38, reflux *n* = 37.

**FIGURE 4:**
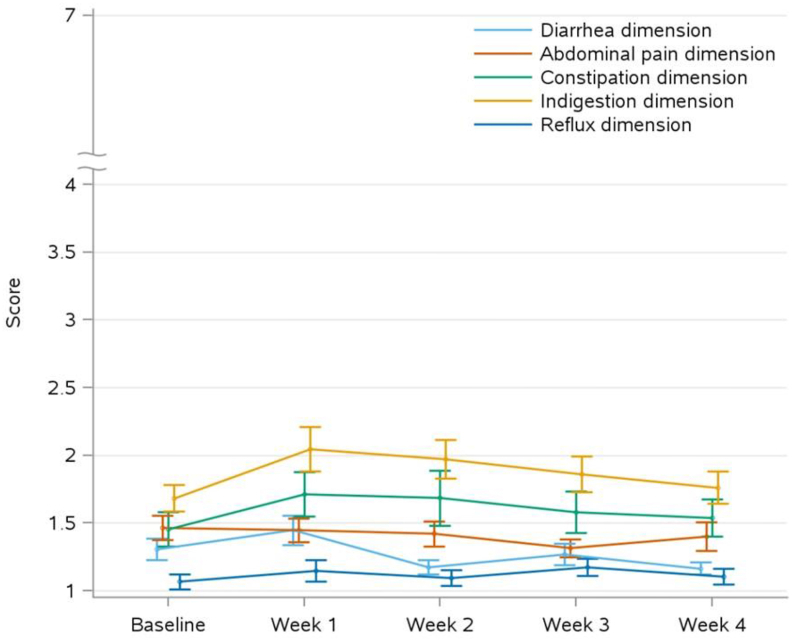
Gastrointestinal Symptom Rating Scale dimension scores per week for all 5 dimensions. Data are represented as mean ± 1 standard error (SE) in the per-protocol data set. Sample size per dimension: diarrhea *n* = 38, abdominal pain *n* = 38, constipation *n* = 36, indigestion *n* = 38, reflux *n* = 37.

Changes in GSRS dimensions remained below their respective MIDs from baseline to all weeks (Figure 3), except for the change in indigestion dimension from baseline to week 1, with least-square (LS) mean and standard error (SE) of 0.4 ± 0.2, where the upper 95% CI (0.0, 0.7) reached the MID of 0.7 (Figure 3D). The findings in FAS are in line with the reported PP results, except that the MID of indigestion was not reached at week 1 [LS mean ± SE of 0.35 ± 0.16 and 95% CI (0.02, 0.68); see Supplementary Figure 2]. Mean GSRS score per dimension ranged from 1.1 ± 0.4 (for reflux, week 2) to 2.1 ± 1.0 (for indigestion, week 1), representing no to minor symptoms (Figure 4). FAS results are in line with reported PP findings (Supplementary Figure 3).

#### Fecal transit type and frequency

The majority of participants reported a normal fecal transit type (75.5% ± 24.7, 71.7% ± 28.9, and 74.1% ± 29.1 at baseline, week 1 and week 4, respectively), followed by slow transit (14.6% ± 21.2, 17.8% ± 25.0, and 18.0% ± 27.7), and fast transit (9.9% ± 17.9, 10.6% ± 21.5, and 7.9% ± 17.7). There was no statistically significant change in the transit types over time for both PP (Table 3) and FAS (Supplementary Table 3). The weekly fecal frequency was 8.0 ± 2.8, 9.4 ± 3.5, and 8.0 ± 3.1 stools per week for baseline, week 1, and week 4, respectively. A statistically significant increase for change in weekly fecal frequency from baseline to week 1 was observed with an LS mean ± SE of 1.4 ± 0.4 and 95% CI (0.5, 2.3), but not from baseline to week 4 (Table 3). FAS analysis showed similar results (Supplementary Table 3).

**TABLE 3:** Change in fecal transit type and fecal frequency, obtained with mixed model for repeated measures for respective weeks to baseline, of the per-protocol data set

	Timepoint	*n*	Timepoint difference (week x – baseline)^1^	
			LS mean	95% CI
Fecal transit type (Bristol Stool Form Scale), slow transit (% total)	Week 1	38	3.2	(‒3.5, 9.8)
	Week 4	38	3.4	(‒5.3, 12.2)
Fecal transit type (Bristol Stool Form Scale), normal transit (% total)	Week 1	38	‒3.9	(‒11.9, 4.2)
	Week 4	38	‒1.4	(‒11.9, 9.2)
Fecal transit type (Bristol Stool Form Scale), fast transit (% total)	Week 1	38	0.7	(‒4.2, 5.6)
	Week 4	38	‒2.0	(‒8.7, 4.6)
Fecal defecation frequency, stools/week	Week 1	38	1.4	(0.5, 2.3)^2^
	Week 4	38	0.0	(‒0.7, 0.7)

Abbreviations: CI, confidence interval; LS, least-square.

1 Timepoint difference between baseline and week 1 or week 4 is reported as the LS mean with 95% CI, obtained with mixed model for repeated measures.

2 Represents statistical significance based on the 95% CI.

#### DQLQ

In the PP data set, the DQLQ scores were 0.2 ± 0.3 at baseline and 0.3 ± 0.5 at week 4. The change in DQLQ score was not significant, with LS mean ± SE of 0.14 ± 0.07 and 95% CI (‒0.001, 0.286). In contrast, in the FAS data set, DQLQ scores were 0.1 ± 0.3 at baseline and 0.3 ± 0.5 at week 4, and showed a small but statistically significant increase from baseline to week 4 with an LS mean ± SE of 0.2 ± 0.1 and 95% CI (0.0, 0.3).

#### Safety evaluation

Fourteen participants who started product intake had ≥1 AE assessed by the investigator as related (probable or possible) to the study product (Supplementary Table 4). No serious AEs were reported. All related AEs were GI in nature and assessed to be mild by the investigator, except for 1 probably related moderate event of constipation, which led to early termination. Most frequently reported related AEs were flatulence and abdominal pain.

## Discussion

This is the first study evaluating GI tolerance following consumption of the FM, a diverse mixture of fibers in proportions similar to those typically consumed in a nutritionally balanced diet, comprising arabinoxylan, β-glucan, pectin, and resistant starch. It was demonstrated that the FM produced SCFAs, whereas moderating gas production in vitro, resulting in a preferential SCFA-to-gas ratio. Healthy adults aged 45‒69 y consuming 6 g/d of FM for 4 wk showed no significant change in GI tolerance symptoms.

### In vitro fermentation

The observed SCFA production of the individual fibers is in line with other in vitro studies [[37], [38], [39]], and higher SCFA production from mixtures of fiber types has also been reported by others [40,41]. One study reported that 2 different mixtures of 5 fibers [arabinoxylan, pectin, resistant starch, konjac glucomannan, and fructo-oligosaccharide; or arabinoxylan-oligosaccharide, resistant starch, chitin-glucan, konjac glucomannan, and galacto-oligosaccharide (GOS)] resulted in higher SCFA production compared with some of the individual fibers, yet when a “super mixture” was made with equal proportions of both mixtures (a total of 10 fibers), SCFA production was similar to the mixtures of 5 fibers [40]. Another study also reported mixture-dependent effects; for example, resistant starch combined with inulin or yeast β-glucan resulted in increased SCFA production compared with inulin or yeast β-glucan alone. However, the addition of GOS to resistant starch did not increase SCFA production compared with GOS alone [41]. Thus, it seems that there is not always synergy when 2 or more fibers are combined, and more is not always better. However, in our in vitro fermentation model, a synergy in SCFA production was observed using FM compared with the individual fibers. Excessive gas production could be perceived as an undesired side effect when consuming fibers [26,27]; therefore, this was interesting to include in the in vitro analysis. Our model showed that FM did not result in higher gas production compared with the individual fibers. These findings could support the addition of FM to a nutritional supplement to complement fiber intake in individuals with insufficient fiber intake.

### GI tolerance study

Building on the in vitro data, GI tolerance of the FM was studied in healthy adults, representing a crucial first step in establishing GI tolerance. Overall, there was no significant or clinically relevant change (i.e., exceeding MID) in GSRS scores for most dimensions, except for indigestion from baseline to week 1. Overall, GSRS scores were low and in line with the general population [31], suggesting good GI tolerance. The indigestion dimension covers, among others, abdominal distension and increased flatulence. Those symptoms are likely indicative of the fibers being fermented in the gut [24,25]. The indigestion MID was only reached for week 1, hence it was considered modest and temporary, probably indicating an adaptation period to increased fiber consumption, which has been described by others [16]. Progressive increases in fiber intake and educating consumers about the occurrence of such GI tolerance symptoms in the first week(s) of increased consumption could help overcome barriers to increased fiber consumption in the broader population. Overall, GI tolerance of the individual fibers of the mixture has been studied previously in healthy adults. For arabinoxylan, 15 g/d for 5 wk did not result in a change in GI tolerance symptoms compared to control [42]; however, higher intake of >25 g/d for 6 wk resulted in increased GI tolerance symptoms compared to control, but the symptoms decreased over the intervention period and remained within acceptable ranges [43]. Acute cereal β-glucan intake of 28 g/d did not result in more GI tolerance symptoms than the background diet [44]; however, 3 g/d consumption for 4 wk resulted in increased diarrhea and abdominal pain symptoms compared to baseline, but symptom scores were lower compared to week 2 [45]. Pectin consumption of 50 g/d for 2 wk resulted in no adverse side effects [46]; however, 10 g/d for 3 wk resulted in a small but significant increase in the GSRS indigestion dimension compared to control, but the score remained within an acceptable range [47]. In addition, 38.5 g/d for 9 d resulted in increased flatulence in healthy males [48]. Resistant starch is well tolerated for 12 wk at a dose of 7.7 g/d [49], and 10 g/d and 20 g/d for 50 d [50]. However, consumption of 7.5 g/d and 15 g/d for 3 wk [51], 12 g/d for 2 wk [52], 20 g/d for 10 d [53], 21 g/d [54] and 15 g/d and 25 g/d for 3 wk [55] resulted in a minor increase in flatulence compared to control and intake of >50 g/d for 1 mo resulted in flatulence and mild abdominal pain [56]. These findings highlight the diversity of GI tolerance responses based on fiber types, dose, and duration of intake. The quantity of individual fibers in the FM does not exceed the amounts described in the human clinical studies reporting GI tolerance symptoms, which could explain the positive GI tolerance observed following consumption of this FM.

The participants consistently reported normal fecal transit type during the study period, which is in line with the general healthy population [57]. The weekly fecal frequency significantly increased from 8.0 ± 2.8 at baseline to 9.4 ± 3.5 at week 1. However, this is in line with normal bowel habits in the healthy population (3‒21 stools per week) [57]. The significant increase in fecal frequency in week 1 could also be a sign of fiber fermentation, which has an impact on fecal biomass, and FM contains physicochemical characteristics to stimulate fecal regularity [2,58]. This could be a short adaptation period because, after 4 wk, there was no significant difference in fecal frequency compared with baseline. Overall, it was considered a positive outcome that normal fecal transit type and frequency were maintained following FM consumption by the healthy adults. The impact of GI tolerance symptoms on quality of life was assessed using the validated DQLQ [35] as an exploratory outcome; in PP, no significant change was observed. Yet, for the FAS data set, a small but significant increase in DQLQ score from baseline to week 4 was observed; however, both scores were close to the minimum score of 0, hence the change was not considered clinically relevant for the healthy participants. GI tolerance symptoms were evaluated using both the GSRS questionnaire and AE reporting, allowing us to capture symptoms through 2 complementary approaches. Importantly, all reported AEs were GI in nature and overlapped with the symptoms assessed by the GSRS, without introducing any additional or unexpected complaints. This reinforces that our dual assessment approach was comprehensive, while confirming that no further GI issues emerged beyond those already captured by GSRS.

### Strengths and limitations

This study has several strengths. First, the human clinical intervention study was preceded by in vitro testing. Self-reported compliance was high, and GI tolerance was assessed using a validated questionnaire. The dose of 6 g/d was selected to address the “fiber gap” in the general population; however, the habitual fiber intake in our study population was higher than that of the average Dutch population. Still, the addition of FM to the habitual diet enabled an additional 27% of participants to meet the recommended intake of 25 g/d at week 1 and 18% at week 4. A recent review on clinical assessment of GI tolerance proposed 6 g/d as a relevant supplementation dose [25]. Higher FM doses may represent an additional strategy to address insufficient dietary fiber intake and could confer physiological benefits; however, the GI tolerance of higher FM intakes should be evaluated in future studies.

A limitation of the current study was the use of a single-arm, noncontrolled design. However, given the elevated sample size and the robust baseline characterization, this approach is considered an efficient design to evaluate GI tolerance in healthy adults. We selected the diarrhea dimension as the primary outcome because it was considered the most clinically meaningful GI tolerance symptom.

### Future research

Studying GI tolerance is a crucial first step to increasing fiber intake in healthy individuals via consumption of FM. Yet future research should explore GI tolerance in more susceptible individuals, for example, with sub-optimal GI health and/or patients with underlying disorders, as FM consumption may have a stronger effect on their GI tolerance and they may benefit from high(er) fiber intake via supplementation with FM. In addition, it is of interest to study the physiological benefits of longer-term FM intake beyond GI tolerance, for example, the impact on the microbiome, metabolic health, satiety, and/or weight management. Different dosages dependent on or titrated for the habitual fiber intake of the study population could represent another opportunity to bridge the shortage in fiber intake from the diet and potentially result in the physiological benefits of FM consumption.

In conclusion, it was demonstrated that 4 wk consumption of 6 g/d FM intake, comprising of arabinoxylan, β-glucan, pectin, and resistant starch, in addition to habitual dietary intake, was well tolerated by healthy adults. This well-tolerated FM may offer a practical means to help individuals who have difficulty achieving the recommended daily fiber intake.

## Author contributions

The authors’ responsibilities were as follows – LD, MK, JvB, ST, JdV-vdB, LH, AvH: designed the research; LD, AB, MK: conducted research; LD, MK: analyzed data; LD, MK, JvB, ST, JdV-vdB, JR, DJ, LH, AvH: interpreted data; LD, MK, AB, JvB, ST, JdV-vdB, DJ, JR, LH, AvH: wrote the article; LH, AvH: had primary responsibility for final content; and all authors: read and approved the final manuscript.

## Data availability

Data described in the manuscript, code book, and analytic code will be made available upon request, pending application and approval.

## Declaration of AI and AI-Assisted Technologies in the Writing Process

During the preparation of this work the authors used Microsoft 365 CoPilot in order to receive suggestions how to improve the readability, and reduce inconsistencies and redundancies as mostly nonnative English writers. After using this tool/service, the authors reviewed and edited the content as needed and take full responsibility for the content of the publication.

## Funding

This study was sponsored and executed by Danone Research & Innovation (Utrecht, The Netherlands).

## Conflict of interest

LD, MK, AB, JvB, ST, JdV-vdB, JR, LH, and AvH are employed by Danone Research & Innovation. DJ report no conflicts of interest.
