# Supplementary material for: A Diverse Fiber Mixture, Reflective of a Nutritionally Balanced Diet, Is Well Tolerated by Healthy Adults
Source: Curr Dev Nutr. 2026 Apr 18;10(6):107695. doi: 10.1016/j.cdnut.2026.107695 (PMC13223861; doi:10.1016/j.cdnut.2026.107695)
Supplement: Multimedia component 1 [file mmc1.docx]

**Supplementary Figure 1:** Participant flow chart. The full analysis set consisted of all subjects treated, i.e., all participants who had consumed study product. The per-protocol data set consisted of all participants from the FAS data set who completed the study without infringing compliance criteria or any major protocol deviations.

**Participants screened**

(*n* = 52)

**Participants enrolled**

(*n* = 43)

**Not enrolled** (*n* = 9)

Reasons for screen failure:

- Not meeting exclusion criteria (*n* = 5)
  - Medical condition (*n* = 1)
  - Use fiber/pre-/pro-/postbiotic dietary supplements (*n* = 1)
  - Food allergies (*n* = 2)
  - International travels (*n* = 1)
- Other (*n* = 4)

**Analyzed PP**

Diarrhea dimension of GSRS: *n* = 38

Abdominal pain dimension of GSRS: *n* = 38

Constipation dimension of GSRS: *n* = 36

Indigestion dimension of GSRS: *n* = 38

Reflux dimension of GSRS: *n* = 37

Reflux dimension of GSRS: n = 37

**Completed**

(*n* = 39)

**Early withdrawals**

(*n* = 4)

**Reasons for exclusion from PP analysis**

- Diarrhea, abdominal pain, and indigestion dimensions of GSRS (*n* = 1)
  - Study product intake in a baseline week (*n* = 1)
- Constipation dimensions of GSRS (*n* = 3)
  - Study product intake in a baseline week (*n* = 1)
  - Use of concomitant medication potentially resulting in an overestimate of a positive effect of the constipation dimensions of GSRS (*n* = 2)
- Reflux dimensions of GSRS (*n* = 2)
  - Study product intake in a baseline week (*n* = 1)
  - Use of concomitant medication potentially resulting in an overestimate of a positive effect of the reflux dimension (*n* = 1)

**Analyzed FAS**

(*n* = 41)

(no data collected *n* = 2)

FAS, full analysis set; GSRS, Gastrointestinal Symptom Rating Scale; PP, per-protocol.

**Supplementary Figure 2:** Change in Gastrointestinal Symptom Rating Scale scores (GSRS) from baseline, presented as predicted changes with 95% confidence interval, derived from a mixed model for repeated measures, of the full analysis set for the following dimensions: (A) diarrhea, (B) abdominal pain, (C) constipation, (D) indigestion, (E) reflux. The horizontal line represents the minimal important difference (MID), a measure of clinical relevance. Sample size for all dimensions: baseline n = 41, week 1 n = 40, weeks 2, 3 and 4 n = 39.


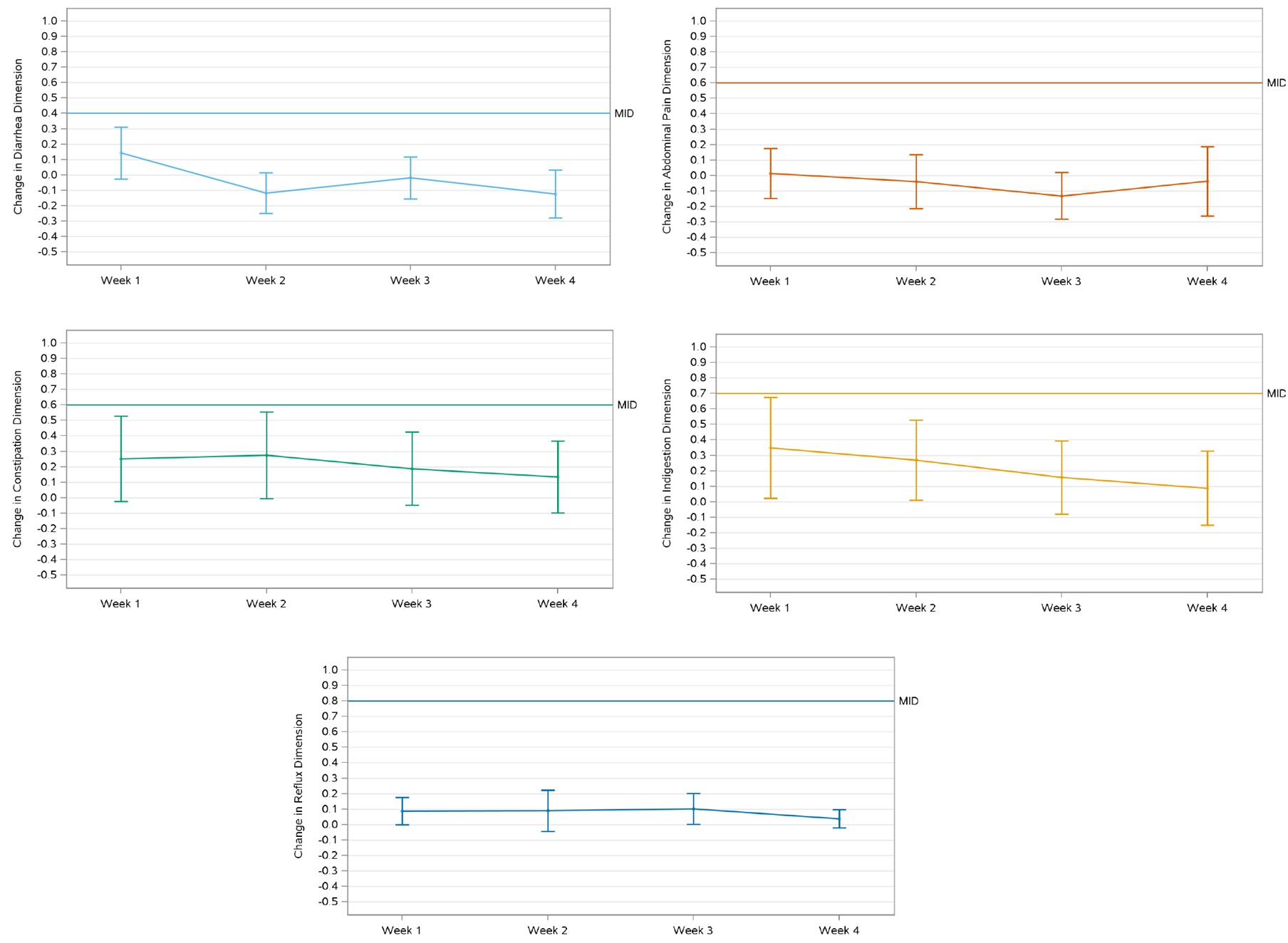


E

D

C

B

A


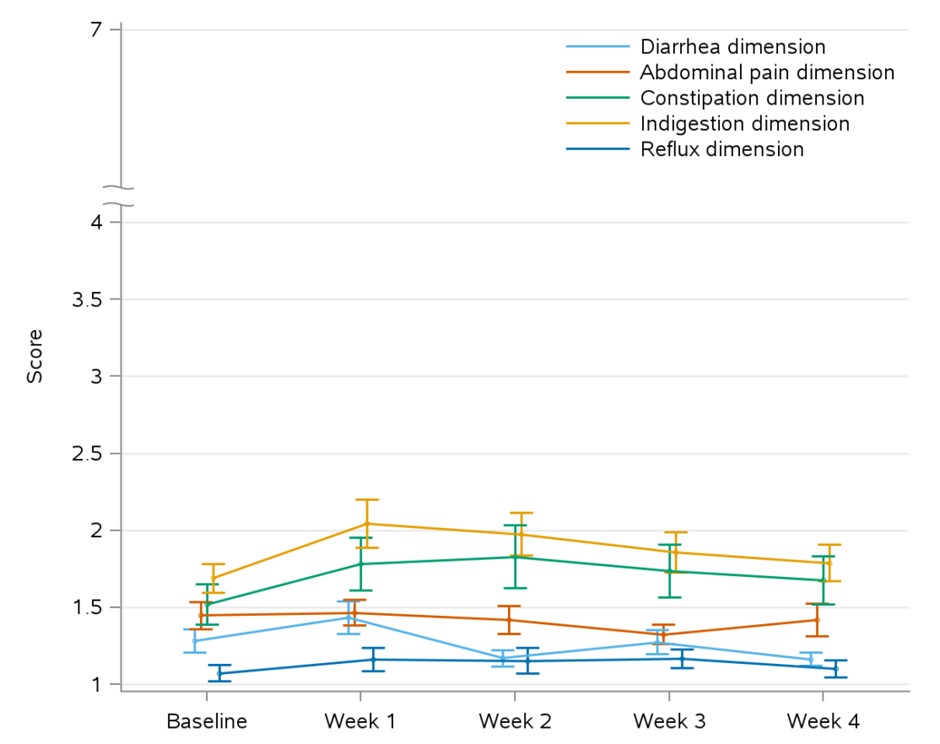
**Supplementary Figure 3:** Gastrointestinal Symptom Rating Scale dimension scores per week for all five dimensions. Data are represented as mean ± 1 standard error (SE), in the full analysis set. Sample size for all dimensions: baseline n = 41, week 1 n = 40, weeks 2, 3 and 4 n = 39.

**Supplementary Table 1:** Exclusion criteria in the gastrointestinal tolerance clinical study

| Presence or prior history of chronic gastrointestinal diseases (e.g., gastric ulcers, ulcerative colitis, Crohn’s disease) or history of abdominal surgery involving resection of bowel (small or large intestine). |
| --- |
| Presence of any other medical conditions where gastrointestinal function is commonly/ likely affected, such as:  Auto-immune diseases (e.g., type I diabetes mellitus, multiple sclerosis, systemic lupus erythematosus).  Eating disorders (e.g., anorexia nervosa, bulimia nervosa, binge-eating disorder).  Any bowel/gastrointestinal dysfunction as sequelae of stroke.  If female participant, irregular bowel habits (i.e., diarrhoea, constipation) and gastrointestinal discomfort (*i.e.,* abdominal pain, indigestion and gastro oesophageal reflux), associated with the menstrual cycle and/or used contraception. |
| Any chronic diseases (including but not limited to hypertension, dyslipidaemia, type 2 diabetes mellitus, thyroid disorders) which are uncontrolled with the use of medication/therapy. |
| Active/ current malignancies except squamous cell carcinoma of the skin or carcinoma in situ. |
| Severe acute liver disease or chronic liver disease. |
| Severe or chronic renal disease or requirement for haemodialysis. |
| Colonoscopy, irrigoscopy or other bowel cleansing, or other surgical procedures related to the GI track (e.g., colon polypectomy, appendectomy) or requiring the use of systemic antibiotics within 1 month^1^ before screening or planned during the participation in the study. |
| Use of enemas, laxatives, antacids, proton pump inhibitors, prokinetics drugs (e.g., metoclopramide, medications that affect motility), digestive enzymes, narcotic/morphine analgesics within 1 week before the screening or intended use during the participation in the study. |
| Use of systemic antibiotics within 1 month^1^ before the screening or intended use during the participation in the study. |
| Use of fiber supplements and other prebiotic, probiotic, and postbiotic supplements within 1 month^1^ before the screening or intended use during the participation in the study, other than the prescribed study product. |
| Any known food allergies or food intolerances. |
| Pregnancy or lactation at the time of study commencement or planning pregnancy during the time of a study. |
| Following a vegetarian or vegan diet. |
| Changes in habitual dietary intake, restriction of food intake and/or following a prescribed diet (e.g., aim to lose weight, ketogenic diet, etc.), within 1 month^1^ before the screening or intended change in habitual dietary intake during the participation in this study.^2^ |
| Extreme physical activity patterns (e.g., marathon training, preparation for a competitive event, a master athlete) within 1 month^1^ before screening or intended change into the extreme physical activity pattern during the participation in the study.^2^ |
| Currently smoking or smoking within 1 month^1^ prior to screening (except for incidental smoking of ≤3 cigarettes/e-cigarettes/cigars/pipes/water pipes per week on average in the last month^1^). |
| Excessive alcohol consumption (men >21 glasses/week, women >14 glasses/week, on average during the last 6 months^1^).^3^ |
| International travels within 1 month^1^ before screening or intended international travels during the participation in the study that might affect compliance with the study protocol and introduce behavioural, lifestyle and environmental changes (e.g., vacations abroad that might change dietary habits, work travels outside the European Union (EU) and the United Kingdom, travel within the EU that affect fecal sample collection and storage, non-regular travels abroad). |
| Drug or medicine abuse in opinion of the investigator. |
| Participation in a research study within 1 month^1^ before the screening or intended participation in another research study while participating in this study. |
| Employees and/or family members or relatives of employees of Danone Research & Innovation. |

^1^ Month is defined as 30 days.

^2^ Participants will be asked by the investigator to assess to the best of their knowledge the changes in their dietary and physical activity practices within 1 month before screening.

^3^ Glass of alcohol is a standard serving per type of alcohol. According to the Centres for Disease Control and Prevention 1 drink is defined as 340 mL of 5% alcohol by volume beer, 142 mL of 12% alcohol by volume wine, 43 mL of 40% alcohol by volume distilled spirits or liquor.

**Supplementary Table 2:** Cumulative production of acetate, propionate and butyrate, and the total sum of those short chain fatty acids (SCFA) in µmol/g fiber after 72 h in vitro fermentation in a semi-dynamic batch fermentation model with a fecal pool of healthy adult donors. Testing blank reference control without fiber, fiber mixture (FM) and the individual fibers thereof, standardized for fiber content of 200 mg.

| **Conditions** | **Acetate** | **Propionate** | **Butyrate** | **Total SCFA** |
| --- | --- | --- | --- | --- |
| Blank | 1675 ± 176.8 | 470.0 ± 35.4 | 300.0 ± 63.6 | 2445.0 ± 275.8 |
| Fiber mix | 7500 ± 70.7 | 1172.1 ± 7.1 | 1110.0 ± 91.9 | 9782.1 ± 14.1 |
| Arabinoxylan | 5050 ± 141.4 | 1430.0 ± 176.8 | 932.5 ± 81.3 | 7412.5 ± 399.5 |
| β-glucan | 5850 ± 707.1 | 740.0 ± 70.7 | 1742.5 ± 378.3 | 8332.5 ± 1156.1 |
| Pectin | 6100 ± 141.4 | 750.0 ± 91.9 | 805.0 ± 7.1 | 7655.0 ± 56.6 |
| Resistant starch | 5050 ± 353.6 | 910.0 ± 261.6 | 1450.0 ± 155.6 | 7410.0 ± 770.7 |

**Supplementary Table 3**: Change in fecal transit type and fecal frequency, obtained with mixed model for repeated measures for respective weeks to baseline, of the full analysis set

|  | |  | **Timepoint difference (Week x – baseline)^1^** | | | |  |
| --- | --- | --- | --- | --- | --- | --- | --- |
|  | **Timepoint** | ***n*** |  | **LS mean** |  | **95% CI** | |
| Fecal transit type (Bristol Stool Form Scale),  slow transit (% total) | Week 1 | 41 |  | 3.1 |  | (‒ 3.0, 9.3) | |
|  | Week 4 | 39 |  | 2.5 |  | (‒ 6.3, 11.3) | |
| Fecal transit type (Bristol Stool Form Scale),  normal transit (% total) | Week 1 | 41 |  | ‒ 4.5 |  | (‒ 12.3, 3.2) | |
|  | Week 4 | 39 |  | ‒ 1.0 |  | (‒ 11.5, 9.5) | |
| Fecal transit type (Bristol Stool Form Scale),  fast transit (% total) | Week 1 | 41 |  | 1.4 |  | (‒ 3.6, 6.4) | |
|  | Week 4 | 39 |  | ‒ 1.4 |  | (‒ 8.0, 5.1) | |
| Fecal defecation frequency, stools/week | Week 1 | 41 |  | 1.4 |  | (0.5, 2.2)^2^ | |
|  | Week 4 | 39 |  | ‒ 0.0 |  | (‒ 0.7, 0.7) | |

^1^ Timepoint difference between baseline and week 1 or 4 are reported as LS mean with 95% CI, obtained with mixed model for repeated measures.

^2^ Represents statistical significance based on the 95% confidence interval (CI).

CI, confidence interval; LS, least-square.

**Supplementary Table 4:** Total adverse events and adverse events probably or possibly related to study product intake as assessed by the investigator. Reporting number of events and number and percentage of participants with at least one event by System Organ Class, of the full analysis set

|  | **Total AE *n* = 41** | | | **Related AE¹ *n* = 41** | | |
| --- | --- | --- | --- | --- | --- | --- |
| **Preferred term**  **(MedDRA 26.1)** | **k** | ***N*** | **%** | **k** | ***N*** | **%** |
| **Any System Organ Class** | | | | | | |
| Any event |  | 28 | 68.3% |  | 14 | 34.1% |
| **Gastrointestinal disorders** | | | | | | |
| Any event |  | 14 | 34.1% |  | 14 | 34.1% |
| Abdominal distension | 2 | 2 | 4.9% | 1 | 1 | 2.4% |
| Abdominal pain | 8 | 7 | 17.1% | 7 | 6 | 14.6% |
| Abdominal pain lower | 5 | 1 | 2.4% | 5 | 1 | 2.4% |
| Abdominal pain upper | 1 | 1 | 2.4% | 1 | 1 | 2.4% |
| Constipation | 2 | 2 | 4.9% | 2 | 2 | 4.9% |
| Diarrhea | 1 | 1 | 2.4% | 1 | 1 | 2.4% |
| Flatulence | 6 | 6 | 14.6% | 6 | 6 | 14.6% |
| Food poisoning | 1 | 1 | 2.4% | 0 | 0 | 0.0% |
| Gastrointestinal sounds abnormal | 1 | 1 | 2.4% | 1 | 1 | 2.4% |
| Gastroesophageal reflux disease | 1 | 1 | 2.4% | 1 | 1 | 2.4% |
| Nausea | 3 | 2 | 4.9% | 3 | 2 | 4.9% |
| Rectal tenesmus | 1 | 1 | 2.4% | 1 | 1 | 2.4% |

¹ Possibly or probably related to study product intake as assessed by the investigator.

k = number of events based on preferred term, *n* = number of participants in analysis population, *N* = number of participants with one or more events, % relates *n to N*.

AE, adverse event; MedDRA 26.1, Medical Dictionary for Regulatory Activities version 26.1
